# Between Scylla and Charybdis: reconciling competing data management demands in the life sciences

**DOI:** 10.1186/s12910-016-0112-6

**Published:** 2016-05-17

**Authors:** Louise M. Bezuidenhout, Michael Morrison

**Affiliations:** Steve Biko Centre for Bioethics, Faculty of Health Sciences, University of the Witwatersrand, Parktown Johannesburg, 2193 South Africa; Egenis Centre for the Study of the Life Sciences, University of Exeter, Byrne House St German’s Road, Exeter, Devon EX4 4PJ UK; Centre for Health, Law and Emerging Technologies (HeLEX), Nuffield Department of Population Health, University of Oxford, Ewert House Banbury Road, Oxford, OX2 7DD UK

## Abstract

**Background:**

The widespread sharing of biologicaConcluding Comments: Teaching Responsible Datal and biomedical data is recognised as a key element in facilitating translation of scientific discoveries into novel clinical applications and services. At the same time, twenty-first century states are increasingly concerned that this data could also be used for purposes of bioterrorism. There is thus a tension between the desire to promote the sharing of data, as encapsulated by the Open Data movement, and the desire to prevent this data from ‘falling into the wrong hands’ as represented by ‘dual use’ policies. Both frameworks posit a moral duty for life sciences researchers with respect to how they should make their data available. However, Open data and dual use concerns are rarely discussed in concert and their implementation can present scientists with potentially conflicting ethical requirements.

**Discussion:**

Both dual use and Open data policies frame scientific data and data dissemination in particular, though different, ways. As such they contain implicit models for how data is translated. Both approaches are limited by a focus on abstract conceptions of data and data sharing. This works to impede consensus-building between the two ethical frameworks. As an alternative, this paper proposes that an ethics of responsible management of scientific data should be based on a more nuanced understanding of the everyday data practices of life scientists. Responsibility for these ‘micromovements’ of data must consider the needs and duties of scientists as individuals and as collectively-organised groups.

**Summary:**

Researchers in the life sciences are faced with conflicting ethical responsibilities to share data as widely as possible, but prevent it being used for bioterrorist purposes. In order to reconcile the responsibilities posed by the Open Data and dual use frameworks, approaches should focus more on the everyday practices of laboratory scientists and less on abstract conceptions of data.

## Background

In 1627 Francis Bacon wrote “[w]e have consultations, which of the inventions and experiences which we have discovered shall be published and which not; and all take an oath of secrecy for the concealing of those which we think fit to keep secret” [1] paragraph 87. Despite its age, the quote is as topical today as on the day it was written, as it exemplifies a perennial concern of scientists: *what constitutes responsible management of data?* In particular, how can be benefits of sharing data be balanced by the possibility that some shared data may have the potential to cause harm?

In the twenty first century, these familiar tensions are being reshaped by a range of developments from the widespread use of information communication technologies (ICTs) to post-9/11 global security concerns. Two key concepts that exemplify these changes are ‘dual use’ and Open Data. The former advocates the careful scrutiny and possible control of published scientific data to ameliorate threats of bioterrorism, while the latter advocates the maximal dissemination of both published and unpublished scientific data to facilitate optimal re-use.

How dual-use concerns might be reconciled with Open Data expectations is of considerable significance for shaping scientific practices and influencing the policies that control research. Indeed, in an increasingly “data-centric” age, understanding how the possible harms of disseminating research should be reconciled with the benefits of increasing openness and access to data are crucial for future scientific development. Moreover, as translational medicine increasingly becomes a driving force in visions of healthcare, better understanding of how translation- especially the ‘T1’ phase of translation of findings from basic science to (clinical) application—can be ethically undertaken is vital. Given that sharing scientific data is regarded as a significant component in fostering translational research both frameworks also have implications for how translation operates in practice.

### Understanding open data and dual-use discussions

Dual-use and Open Data concerns have recently become a key topic in funding applications. When scientists apply for funding, they are regularly confronted with statements that reflect specific aspects of data management, vis Open Data or dual-use concerns. These are often very similar to the ones below:

*“It is the responsibility of institutions in receipt of Wellcome Trust funding to ensure that any risks that research could be misused for harmful purposes are managed in an appropriate manner. Please confirm that you have considered whether your proposed research could generate outcomes that could be misused for harmful purposes.”*^1^

*“Our position statement on data management and sharing requires that all of our funded researchers maximise the availability of their research data with as few restrictions as possible.”*^2^

At first glance synthesizing these two requirements into a holistic data management plan may not seem so difficult to resolve. Could scientists not simply balance their ethical duties by making data freely available except when there are compelling reasons not to do so? After all, a similar approach has been applied to disseminating sensitive data concerning human subjects [2, 3]. Indeed, considered in the abstract (and using prevalent examples of data management such as those from clinical trials or nuclear research), the ethical duties of an individual scientist may seem relatively straightforward, but once consideration turns to operationalization it is much less obvious what these responsibilities might mean in terms of the everyday practices of routine scientific work.

Even if taken at face value, it is evident that much of the responsibility for data management is placed on the individual researcher who needs to make strategic decisions not only about benefits and harms, but also about what data to share, how and why. This places considerable expectations on scientists not only practically, but also from an ethical perspective—as it is often argued that scientists hold some responsibility regarding the outcomes of their research (for example [4]).

The provision of ethics education for scientists is not without its challenges and limitations. Although scientists may receive training in the ‘responsible conduct of research’ they are generally not well-versed in how to reconcile competing policy demands, and ethics training within the scientific community remains patchy and unstandardized [5]. Without considerable further efforts to improve ethics training at undergraduate and postgraduate levels, it is difficult to see how the ethical responsibilities of data management can be reasonably expected from the scientific community.

Moreover, as discussed in this paper, making such expectations of scientists is further problematic for two important reasons: first, that discussions on data management tend to be articulated at the level of *research directions* rather than the individual data generation and dissemination activities that make up the daily work of scientists. Second, that Open Data and dual use discourses differ considerably in their perspectives of what constitutes “data”, “dissemination” and by extension, translation, making easy reconciliation of responsibilities highly problematic.

In order to illustrate this, a brief account of the dual use and Open Data movements will be presented. In particular, attention will be drawn to what are prioritized as “data”, under what channels they move, and where the lines are drawn between important and “unimportant” data and dissemination pathways. This will be used to draw out the different framings of scientific responsibility in the two frameworks. The remainder of the paper will emphasize the limitations of each framework, with reference to translation, and offer suggestions towards reconciling and uniting these disparate discussions. It should be noted that both Open Data and dual use frameworks cover scientific data from a range of fields and are not explicitly or specifically concerned with data derived from or concerning human subjects. The issues around security and dissemination of sensitive human data are addressed in depth elsewhere and are therefore not significant part of the analysis that follows.

### Dual-use: addressing the threat of bioterrorism

The September 11th attacks on the World Trade Centre in 2001 and the subsequent posting of Anthrax-infected letters through the US mail service produced a heightened concern with terrorism in many nations. In particular the Anthrax attacks drew attention to the potentially destructive role that scientific research could play in future terror attacks—heralding growing concerns about “bioterrorism”.

Led by the US, many governments started to question whether the very information that was being generated for beneficial research could also be misapplied for destructive purposes. These concerns came to be known as “dual-use concerns” [6] and have since become topics of considerable discussion—particularly within the life sciences. The securitization of dual-use discussion has been highly influential and caused the issue to be: “moved out of the sphere of normal politics into the realm of emergency politics, where it can be dealt with swiftly and without the normal (democratic) rules and regulations of policy making” ([7], p. 748).^3^

From the outset, the dual-use discussion in the life sciences focused on the potential misapplication of research results. The appearance of several high-profile journal articles detailing research that was regarded as having considerable potential for such misapplication further reinforced this focus. These research articles, such as the synthesis of a super-virulent mousepox virus [8], the resurrection and sequencing of the 1918 Spanish Flu virus (For an overview see [9]) and the de novo synthesis of the polio virus [10] have all played key roles in the development of the dual-use discussion and have been highly influential as case studies for ascertaining how dual-use threats could be ameliorated.

In the first decade of the 21st century a number of highly influential reports were issued in order to delineate these concerns within the life sciences. The first, issued by the US National Research Council (NRC), was entitled *Biotechnology Research in an Age of Terrorism* [11] and focused attention to a list of “experiments of concern” (dual-use research of concern: “DURCs”^4^)—research with considerable potential for weaponization. A second, from the National Science Advisory Board for Biosecurity (NSABB), *Globalization, Biosecurity and the Future of the Life Sciences* [12], advocated the establishment of a “web of prevention” model taking into consideration the multifaceted actions needed to address dual-use concerns. Both these reports focused on the risks posed by the possible misuse of *published* research [13].

Subsequent dual-use discussions have been heavily influenced by these reports, and have predominantly focused on the possible dangers posed by published research—particularly in emerging fields such as nanotechnology and synthetic biology [7]. As a result, much of the discussion has focused on whether results should be published or not. In 2003 the editors of 23 journals—including *Nature* and *Science*—proposed the establishment of “pre-publication review” of potentially harmful publications [14]. This “extra tier” of review for biosecurity concerns has subsequently been employed by a considerable number of other journals, although as yet no publications have been rejected for security purposes.^5^ Requirements to identify dual-use concerns have also rapidly started to appear as components of the grant application forms employed by many high profile funders such as the NIH and Wellcome Trust. While there are, of course, problems associated with identifying the possible harms arising from a specific research project, there has been considerable support for further development in this field [13].

Dual-use discourse thus makes use of very specific interpretations of “data” and “dissemination”. The very influential reports published by the NRC [11, 15] and NSABB [12] clearly direct attention towards *published articles* [7, 13]—and away from so-called ‘raw data’, repositories and databases. Similarly, the focus is predominantly towards *peer-reviewed journals—*and away from informal dissemination pathways, crowd research and discussion forums. The important role allocated to DURCs as both thought experiments and the basis for policy development is evidence of this distinction.

The framing of precaution and control has been highly influential in not only the dual-use discourse, but also the codes of conduct, regulations, data statements and legislation resulting from it that pertain to individual responsibility. A 2007 publication by the NSABB noted this responsibility, saying that: *“[i]ndividuals involved in any stage of life sciences research have an ethical obligation to avoid or minimize the risks and harm that could result from malevolent use of research outcomes”* ([16] p. 9) Such statements were recently lauded in a paper by Selgelid that noted: *“[a] virtue of much of the emerging dual-use ethics literature is that it takes seriously the idea that individual scientists have significant responsibilities regarding the prevention of harm resulting from malevolent use of their research”* ([17], p. 30).

These responsibilities for scientists, as summarised by Kuhlau et al, include: *“the duty not to publish or share sensitive information, […] the duty to oversee or limit access to dangerous materials, [and] the duty to report activities of concern”* ([4], pp. 483–486). This sentiment of vigilance and control is, of course, extended beyond the scientific community to the multiple stakeholders involved in biosecurity. This is recognized by numerous funders and governmental bodies, for example: *“[t]he BBSRC, MRC and WT consider that in order to address these legitimate concerns, it is important that appropriate processes exist at institutional, national and international levels for the review and oversight of research that could potentially be misused to cause harm. The funders have stressed the need for researchers to identify, consider and report cases of potential concern”* ([18], p. 2).

From the quotes above it is evident that the responsibilities of scientists are assumed to pertain to “data” generally. Nonetheless, as the representation of “data” as research publications in academic journals and DURCs are most influential in problematizing these discussions, these discussions on extended responsibilities are influenced by this legacy. In consequence (as evident above), discussions on responsibility seem to portray data as discrete packets of information that travel as units along linear pathways. 6 This lends itself to the idea of scientists as “gatekeepers”, “a surveillance network” [19] or the “first line of defence” [20] in dual-use control. Scientists, in this framing, thus may act responsibly by alerting the wider community to items of data that may be diverted for nefarious purposes, or by blocking the dissemination of that data.

This framing of “data” is, of course, very specific, and there has already been discussion within the dual-use community about how effectively any sort of control of information can hope to be—particularly in the increasingly digital world. Nonetheless, despite these criticisms, dual-use discussions have yet to critically engage with other framings of “data” and their movement—as will be elaborated on below. Indeed, how dual-use responsibility for pre-publication data (particularly sequence data), methodologies, and extended metadata are understood remains unclear. Similarly, how scientists’ act upon dual-use responsibilities outside of the formal avenues of data dissemination require much further investigation.

### Open Data: ensuring the maximal re-use of research data

The move towards Open Data (and Open Access) has been facilitated by advances in ICTs. Digital and internet technologies have removed many of the traditional barriers to the widespread dissemination of scientific information such as distance and speed of transmission [21]. Improvements in computing power and automation have also changed the scale of scientific data generation. In genomics, for example, high-throughput sequencing technology has heralded a transformation from “small-scale, single-molecule, laboratory-based research to large-scale, in silica research, in which tens of thousands of genes, transcripts, and/or proteins can be studied simultaneously” ([22], p. xi).

The *push* for greater sharing of scientific data however, comes primarily as a result of particular policy goals. More widespread availability of scientific data is seen as having the potential to improve the reproducibility of experiments and increase transparency, which in turn has been regarded as a mechanism to safeguard public trust in science [23, 24]. Another major thrust of the Open Data movement is the idea that making scientific data ‘maximally available’ will in turn maximise the use and reuse of this data in ways that will increase both scientific and economic productivity and allow states to increase the likely returns on their investment in publicly funded science [23, 25]. This conception of widespread data reuse ties in with accounts of ‘big data’ that emphasise the productive possibilities of combining large data sets to yield novel scientific insights [26]. Increased data sharing is also a necessary corollary of a move away from a “one-scientist-one-project” approach towards larger, geographically distributed, collaborative projects in science with greater potential to address ‘global challenges’ [27].

In this respect the Human Genome Project (HGP) has been an important template for the Open Data movement [21]. Although there were earlier examples of data sharing infrastructures such as the NIH-funded GenBank database of SNP sequences founded in the 1980s, the HGP produced the high-profile Bermuda principles for (genomic) data sharing on which subsequent data dissemination agreements such as the Toronto statement are based [28]. The different laboratories working on the publicly funded HGP agreed to publish each sequence fragment larger than 1000 base pairs within 24 h of generation to a publicly accessible website [28] This set a basis for the pre-publication dissemination of data which has since been adopted by funders such as the NIH, Wellcome Trust and the Research Councils of the UK among others [2]. Although the Bermuda principles for data sharing were developed in the particular context of genomic data in the HGP, by the time of the Toronto statement in 2009 the remit has expanded to include prepublication dissemination of “large reference data sets in biology and medicine that have broad utility” including “chemical structure, metabolomic and RNA interference data sets, and […] annotated clinical resources (cohorts, tissue banks and case-control studies)” ([28], p. 168). Of course sharing human data, especially sensitive data such as medical and genomic information, places limitations on data sharing due to privacy requirements [2, 3]. Access to human genomic data through resources such as dbGaP involves governance systems based on managed access rather than completely free sharing of data. However, as noted above, human data and the particular privacy and access concerns associated with it are not the primary focus of this paper, which is concerned with policies promoting the sharing of scientific data more broadly.

The specificities of Open Data requirements vary by funder, but the underlying ethos is that scientists have a responsibility to make research data “openly available to the maximum extent, and with as few restrictions as, possible, through deposit in digital repositories” ([29], p. 51). The emphasis on prepublication data treats observations, recordings and measurements (made in the course of a scientific experiment or otherwise) as research outputs in themselves. Unlike their deployment in scientific publications, these pre-publication data are seen to have value separate from their use as evidence to support a particular scientific claim [26]. These data sets are regarded as generative of new knowledge, both in isolation and especially when combined with other data sets [26, 29]. Thus in Open Data discussions data are increasingly framed as the basis of both scientific practice and knowledge, prompting claims that, in effect, ‘science is data and that data are science’ ([30], p. 649).

Open Data also marks a significant shift from traditional models of what dissemination means for scientists. Where previously scientists might have decided to share data sets with selected others on the basis of personal acquaintance or reputation, Open Data policies have transformed the issue from ‘whether to share’ to ‘how to share’ [2]. In Open Data, at least from the funder’s perspective, databases and repositories have replaced journals as the dissemination infrastructure of choice. These infrastructures are designed to place data ‘out there’ for reuse and reinterpretation. The anticipated maximisation of production of new knowledge is understood to require a corresponding minimisation of control over access to data sets retained by individual scientists and groups. The responsible scientists’ duty in Open Data terms is therefore to disseminate as much as possible as quickly as possible by placing their data in such repositories with minimal barriers or restrictions to access and reuse.

### Data in translation

The particular understandings of what constitute ‘data’ and how data move (data journeys) enshrined in the Open Data and dual use discussions both have implications for how translation is understood to occur. Open data discussions tend to regard even pre-analysis data (so-called ‘raw’ data) as fundamentally generative of scientific knowledge, as though translation was largely a matter of analysis. However, scientific data do not ‘speak for themselves’; they require interpretation to render them useable and, indeed translatable. This is demonstrated in practice by the requirement for metadata; information about the circumstances in which scientific data was collected or generated, including the tools and methods used, the purposes for which the data was originally collected and the circumstances under which collection occurred to enabling the use and reuse of data [31, 32]. This kind of contextual information also allows scientists to make judgements about the quality of data when deciding whether or not to use data produced by others [33].

Published journal articles are more likely to contain details of the methods and tools used in generating a set of scientific findings. However, it is still a mistake to consider a journal article a self-contained ‘recipe’ for (re)producing a particular scientific finding as the dual use literature is in danger of doing. It is rare that scientific journal articles report all of the contingencies and uncertainties encountered during an experiment or piece of fieldwork, rather representing a formalised, stylised account of events produced in accordance with the publication culture of science [34]. It also requires infrastructure, equipment, expertise, tacit knowledge and practical know-how to reuse data whether pre-analysis or post-publication. These elements of translation are not adequately addressed by either Open Data or dual use discussions.

### Opposing ethical responsibilities, or a need to refocus on practice?

The dual-use and Open Data discourses evolved for different purposes from different policy communities. This situation is exacerbated by the fact the two policy communities rarely engage with one another. Open Data literature generally does not unpack, let alone address dual use concerns, and vice versa. Nonetheless this in itself should not mean that the two positions are incommensurable and that a unified, holistic approach to responsible data management cannot be achieved. Two key concerns, however, stand in the way—first, that the vocabulary and concepts used to discuss data are markedly different in the dual-use and Open Data discussions. The two discussions make use of different interpretations of what constitutes “data”, its value independent of experimentation, and how it moves between research contexts, which necessarily shapes how actions of sharing data are envisioned [32]. Second, that the manner in which data are dissemination are discussed in both fields of discourse is often abstracted and removed from the daily practices of scientific research. How and when to share data in manners that embody these various responsibilities remains unclear.

It is important to note that these situations may not be “true” ethical problems, but instead “wicked problems” in that they are difficult to solve because of incomplete or contradictory requirements, rather than inherent conflict [35]. Nonetheless, the inability for scientists to act according to all the responsibilities assigned to them can cause ‘ethical erosion,’ [36, 37], meaning that the sustained inability to act according to the ethical expectations set out in regulations, policy or teaching may cause scientists to cease to see value in the ethics guidelines and act unethically [38]. These issues are thus very important to consider in the context of responsible conduct of research.

## Discussion

### Considering definitions of “data”

Open Data policies have tended to define data as a-contextual, readily exchangeable ‘units’ of information. This type of pre-publication data is sometimes regarded as ‘raw’ data. The expression is something of a misnomer as even data points deposited wholesale in a database will be subject to some sort of organisation and annotation designed to ‘make sense’ of them. A suitable alternative description might be ‘pre-analysis’ data, as it describes data that have been ordered and standardised to some degree but which has not yet been interpreted and interrogated to produce the results that would form the basis of a scientific publication.

Dual-use literature, by contrast, focuses primarily on data that has been extensively collated and analysed, especially when organised in the form of peer-reviewed journal articles. Both of these interpretations can easily become too abstract—particularly for individual scientists. In a world where the quantity of research data is increasing exponentially it is often difficult for individual scientists to understand their roles—and responsibilities—in the ethics of data production [39].

In this respect, large scale genomic (and proteomic) data sharing projects like the HGP are potentially poor models on which to base standards for scientific data sharing as they involve large amounts of highly standardised, homogeneous data which is actually in contrast to many areas of the life sciences [21]. Social scientists studying the data practices in the life and physical sciences have generally taken a broader approach to defining what counts as ‘data’ [21, 33]. In a series of case studies of data use in different life sciences domains, the Research Information Network [21] found that scientists combine multiple, heterogeneous data types such as genomic profiles, scans and image data, medical histories, taxonomic classifications, transgenic organisms, lab books, published papers, protocols, Standard Operating Procedures, information from public and controlled access databases, specialist wikis, advice from colleagues, conference proceedings, laboratory demonstrations, tool kit instructions, mathematical algorithms, statistical software programmes, fieldwork, and readings from experimental apparatus. These different information sources are combined in multiple, iterative stages from the initial planning of an investigation to the journal article submitted for publication. Thus the scope of information required for scientific practice is broader and more heterogeneous than that commonly envisaged by either Open Data or dual-use policies.

Refocusing on what happens “on the ground, in the lab” might then be a way of gainfully integrating these different discussions. . If discussions on data responsibility developed a holistic view of daily data production that takes into account the entire range of data types and engagement activities—from data generation, storage and curation to dissemination and re-use—it would not only assist scientists in understanding their roles but also eliminate some of the confusion inherent in the terminology employed by different dialogues. What is vital for such a discussion, however, is a careful re-examination of the current ways in which “data” are discussed.

### Defining dissemination: data movements

In recent years authors such as Leonelli have drawn attention to the important role of “data journeys” within the new data-centric research paradigm [26, 40] The ideals of the Open Data movement, she points out, can only be realised if movement of data is facilitated [26]. Similarly, the threats of dual-use arise when data is moved from one context to another—between institutions or communities. How—and what—data moves remains a topic of intense discussion. Leonelli suggests ([26], p. 6) that: “*the vast majority of scientific data generated in the second half of the 20th century have only been accessed by small groups of experts; and very few of those data, selected in relation to the inferences made by the scientists who analyzed them, have been made publicly available through publication in scientific journals*”. What is important in this observation is twofold. First, that publications account for only a small amount of the data that are generated, and second that scientists are highly influential in deciding what data are shared and what not.

Large databases are increasing the mobility of data, although certain types of data, such as genome sequences, are better served by online resources than others. In addition to databases, the internet also offers an increasing range of innovative platforms for sharing scientific data including personal websites, e-books, discussion forums, email lists, blogs, wikis, videos, audio files, RSS feeds and P2P file-sharing networks [41]. These platforms, as well as so-called “altmetric” initiatives—crowdsourcing, social networking and so forth—mark a significant departure from both the formal, peer-review approach of journal publication which dual use discourses focus on and the databases that are at the heart of Open Data policies. Moreover, increasing innovations in data movement pathways are allowing data to move between disciplines, communities and from researchers to the public in previously unprecedented ways.

Scientists, in their data sharing activities thus have to contend with deciding what data to share, and what methods, including which ICT platform(s), should be employed to share it [21, 32, 42]. These options can be thought of as particular data dissemination pathways. Deciding on a pathway involves scientific and logistical concerns, but also has ethical consequences. These ethical issues relate to who can benefit from the pathways—and who can exploit them. Considerations that need to be taken into account when assessing possible dissemination pathways include the cultural and linguistic assumptions underlying their design [42], the ICT resources necessary to exploit them, the cost of using them [41], and their integrity from safety and security perspectives.

Thinking about data journeys is thus important for both Open Data and dual-use discussions. In particular, it highlights the “messiness” inherent in modern data sharing, and the important role that scientists play as not only data generators, but also *selectors* of the data that are released online and *where* it are released to. This necessitates that the ethical responsibilities of data management amongst scientists be revisited. If the structures are in place to facilitate it, there is no particular limit to how data can move across communities or institutions. The responsibility of scientists must therefore be located in the small data transactions of daily research. Scientists thus need to recognise the ethical import of each data transaction—no matter how small or routine. Being critically aware not only about *what* is shared, but *where* is a vital component of ethical behaviour.

### “Micro-movements”: Rethinking responsibility for scientific data

The current literature examining data in scientific research highlights two important considerations: that what constitutes “data” is ever increasing, as are the ways in which these “data” move through the online environment. Moreover, the fundamental role that the individual scientist plays in these two considerations becomes evident when they are critically assessed. Individual scientists, it can be suggested, are in control of *what to use/share, and how to use/share it.* Indeed, all downstream data actions are dependent on these initial decisions made by individual scientists.

The RIN study on data sharing practices asked scientists to fill in “data journals”, mapping out what data moved in and out of their research context on a daily basis [21]. What was evident from this study was the myriad of small data transactions-or “micro-movements” that occur on a daily basis. The idea of “micro-movements” of data represents the daily activities that the individual scientist encounters that involve data being moved on- or off-line, and across the myriad different data distribution channels outlined above. These movements are controlled not only by regulation, tradition or expediency, but also, as discussed previously, by the tacit knowledge, personal preferences and prejudices of the individual scientist.

A focus on data micro-movements suggests a way to balance dual-use and Open Data concerns and to bring them together in a way that lays the foundations for a more contextually-sensitive approach to discussions on responsible conduct of research. Focusing on the data interactions that happen within the daily research activities of a specific laboratory context removes much of the abstract nature of both dual-use and Open Data discussions. It offers a way to discuss ethical responsibilities in a manner that relates to the agency of individual scientists and of scientific research groups, something which is often crucial in stimulating discussions on responsibility [43]. For scientists this responsibility can be framed according to the following requirements:

- That the individual scientist assesses where the data are coming from—does the origin of the data raise concerns?
- That the individual scientist considers where the data are going to—does it increase the potential for beneficial re-use?
- That the individual scientist considers whether the future location of the data is ethically sound—what structures are in place to uphold the integrity of the data and ameliorate harms?

For Open data and dual-use advocates, the requirements are twofold; first to come together to produce shared guidelines on responsible data management for scientists, and second, for those guidelines to focus on the everyday data practices of scientists rather than abstract conceptions of “data”. In particular it would be beneficial for updated guidelines to provide advice on how to assess whether particular types of data are suitable for sharing on particular types of platform. As a move from the general to the specific it would even be worth considering what characteristics of the data in question and of the possible dissemination methods are most ethically relevant, and practical to assess when scientists are making decisions about what to share and how to share it.

Finally, the level of autonomy and agency attributed to the individual scientist has the potential to affect the amount of responsibility that can be designated to them—but also to how responsibility is framed collectively. This is especially important considering the increasing complexity and scope of life science research which has led to the evolution of a highly collaborative, group-based research community in which each scientist contributes but does not control the entirety of a research project [44, 45]. In framing collective and individual responsibilities for data micro-movements, the work of May [46, 47] is relevant. May proposed that an action was legitimately collective if the individuals in question are related to each other so as to enable each to act in ways that they could not manage on their own. The common moral element allows making decisions self-consciously. Through this, each member of the group comes to have the same intention, either reflectively or pre-reflectively (shared interests and attitudes, solidarity) ([46], p. 64). Thus, while scientists have individual responsibility for the “micro-movements” of their data, there is an element of collective, negotiated responsibility due to the combined actions necessary to construct the pathways along which data moves.

## Concluding comments: teaching responsible data management

This paper reviews the challenges posed for scientists by the conflicting models of responsible scientific data sharing envisaged by Open Data and dual use frameworks. Contemporary ethical and policy discussions on data management are disparate and use differing interpretations of key concepts. Moreover, these discussions—by virtue of their abstracted, global perspective—often make it difficult for individual scientists and scientific groups to interpret these requirements in ways that are meaningful for everyday scientific practice at the local level.

Instead it is proposed that a new focus on individual responsibility to discussions on ethical data management that emphasise the plurality of data and data usage in science and highlight the wide range of data interactions that individual scientists have on a daily basis. We feel that teaching data management from the perspective of “micro-movements” is valuable for future ethics pedagogy as it necessitates that scientists consider not only the wide range of data that they are potentially sharing as of equal value for future re-use, but also be cognizant of the structures of where data has come from or is potentially going to. This will necessitate that the data sharing structures be carefully considered from an ethical perspective—including issues such as access, egalitarianism, security and benefits.

As scientists are already careful and critical about who they micro-share with—both as receivers and donors of data [48, 49]—a focus on micro-movements will be strengthened by tapping into the informal cultures, tacit knowledge and personal preferences that already exist to govern these behaviours. The possibilities for incorporating such teaching into current science curricula are myriad—from training mentors and supervisors to facilitate more attention to these issues in laboratory contexts, to incorporating these issues into discussion on data generation and management within undergraduate courses.

## Ethics approval and consent to participate

Not applicable.

## Consent for publication

Not applicable.

## Availability of data and materials

Not applicable.

## Abbreviations

BBSRC: Biotechnology and Biological Sciences Research Council (UK)
BTWC: biological and toxin weapons convention
DURC: dual use research of concern
HGP: human genome project
ICT: information communication technologies
MRC: Medical Research Council (UK)
NRC: National Research Council—(US)
NIH: National Institutes of Health (US)
NSABB: National Science Advisory Board for Biosecurity
SNP: single nucleotide polymorphism
WT: Wellcome Trust (UK)

## Footnotes

1 For example, this is question 11 off the Wellcome Trust small grant application. This wording is similar in most other funding applications from the Trust and prevalent in other funding bodies’ application forms as well. http://www.wellcome.ac.uk/stellent/groups/corporatesite/@msh_grants/documents/web_document/wtp059821.pdf (accessed 13/05/2016).

2 See the Wellcome Trust’s data sharing policy at http://www.wellcome.ac.uk/About-us/Policy/Spotlight-issues/Data-sharing/Guidance-for-researchers/ (accessed 13/05/2016).

3 Van der Bruggen ([7], p 749) identifies a number of different drivers for this securitization aside from the 9/11 terrorist attacks. He suggests that the increasing focus of the Biological and Toxin Weapons Convention (BTWC) on bioterrorism, the emergence of new infectious diseases such as AIDS, SARS and Bird Flu, rapid globalization, and increasingly international terrorist groups such as al Qaeda have all contributed to the emergence of this discourse.

4 These experiments of concern were identified as research that could:

1. Enhance the harmful consequences of a biological agent or toxin.
2. Disrupt immunity or the effectiveness of immunization without clinical and/or agricultural justification.
3. Confer to a biological agent or toxin, resistance to clinically and/or agriculturally useful prophylactic or therapeutic interventions against that agent or toxin or facilitate their ability to evade detection methodologies.
4. Increase the stability, transmissibility, or the ability to disseminate a biological agent or toxin.
5. Alter the host range or tropism of a biological agent or toxin.
6. Enhance the susceptibility of a host population.
7. Generate a novel pathogenic agent or toxin, or reconstitute an eradicated or extinct biological agent.

5 Indeed, there have been concerns about the efficacy of employing censorship to control biosecurity concerns. It has been suggested that this approach not only undermines the openness of science, but also the trust and confidence that the international community has in scientific research [13].

6 Of course this is a simplification of the representation of data in dual-use discourse, but nonetheless valid for the argument we develop in this paper.
